# Supplementary material for: Discovery and pharmacophoric characterization of chemokine network inhibitors using phage-display, saturation mutagenesis and computational modelling
Source: Nat Commun. 2023 Sep 16;14:5763. doi: 10.1038/s41467-023-41488-z (PMC10505172; doi:10.1038/s41467-023-41488-z)
Supplement: Supplementary file 4 — Source Data [file 41467_2023_41488_MOESM4_ESM.zip › SourceData/scripts/xNNKs_20230808183658.tar!/xNNKs/analysis/heatmaps/SV_HD2X_cationic_heatmap.pdf]

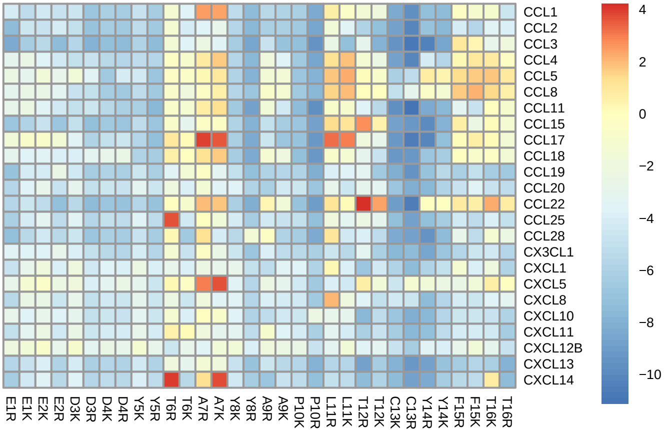
CCL1
CCL2
CCL3
CCL4
CCL5
CCL8
CCL11
CCL15
CCL17
CCL18
CCL19
CCL20
CCL22
CCL25
CCL28
CX3CL1
CXCL1
CXCL5
CXCL8
CXCL10
CXCL11
CXCL12B
CXCL13
CXCL14
E1R
E1K
E2K
E2R
D3K
D3R
D4K
D4R
Y5K
Y5R
T6R
T6K
A7R
A7K
Y8K
Y8R
A9R
A9K
P10K
P10R
L11R
L11K
T12R
T12K
C13K
C13R
Y14R
Y14K
F15R
F15K
T16K
T16R
4
2
0
−2
−4
−6
−8
−10
